# RETRACTION: Exosomes Released from CaSR‐Stimulated PMNs Reduce Ischaemia/Reperfusion Injury

**DOI:** 10.1155/omcl/9769386

**Published:** 2026-04-22

**Authors:** Oxidative Medicine and Cellular Longevity

RETRACTION: T‐Y. Zhai, B‐H. Cui, Y. Zhou, et al., “Exosomes Released from CaSR‐Stimulated PMNs Reduce Ischaemia/Reperfusion Injury,” *Oxidative Medicine and Cellular Longevity*, 2021, 3010548, https://doi.org/10.1155/2021/3010548.

The above article, published online on 12 January 2021 in Wiley Online Library (wileyonlinelibrary.com), has been retracted by agreement between the authors; the Journal Editor‐in‐Chief, Jeannette Vasquez‐Vivar; and John Wiley & Sons Ltd.

The retraction has been agreed due to concerns identified in the figures. The authors initially requested a correction for Figure 2d, which erroneuously duplicated the Western blots from Figure 5c due to a mistake introduced in the production stage. Further investigation by the Publisher also identified an area of overlap between the H/R + NPS PMNs + GW4869 flow cytometry plot in Figure 4b, and the H/R + CIN PMNs EX0s flow cytometry plot in Figure 5b.

After requesting clarification from the authors regarding the flow cytometry concerns, they confirmed that the error was introduced during the assembly of the Figure. They could not provide the flow cytometry raw data, and requested retraction. The editor has lost confidence in the results presented, and so agrees with all parties to retract the article.

